# 

**Published:** 1895-02-09

**Authors:** 


### CONTENTS.

**The Metropolitan Asylums Board and the Anti-Toxin Treatment** - 323

**Tracheotomy** - 324

**The Study of Man.** VIII.—Decorative Art - 325

**The Disposal and Treatment of the Insane** - 326

**Notes and News** - 328

**Medical Progress and Hospital Clinics:**

Head Injuries - 329

PROGRESS IN MEDICINE.—Diseases of the Intestines - 330

PROGRESS IN SURGERY.—General Surgery - 332

PROGRESS IN OPHTHALMOLOGY - 333

NEW DRUGS AND PREPARATIONS - 334

NEW APPLIANCES AND THINGS MEDICAL - 334

**Annotations.**—Cold: Healthy and Unhealthy—Rural Sanitation and the Unemployed—Methuselah: An Apologia - 327

**The Institutional Workshop:**

HOSPITAL ADMINISTRATION.—Hospital Sunday Amenities—Assistant Physicians to Provincial Infirmaries—The Reports of Hospitals—The Planning of Hospitals - 335

FOREIGN HOSPITALS - 336

THE STORY OF THE INSANE FROM YEAR TO YEAR - 337

PRACTICAL DEPARTMENTS - 337

HOSPITAL FESTIVALS AND MEETINGS - 338

**Scraps and Gleanings** - 338

**The Book World of Medicine and Science** - 339

**The Student of Medicine** - 339

**EXTRA SUPPLEMENT.—THE NURSING MIRROR.**

**News from the Nursing World.**—Our Princess—The Collecting Box—Colney Hatch Asylum—Lewisham Infirmary—The Central Sick Asylum—A Nurse for Knottingley—Andover Cottage Hospital—Are Advertisements Tabooed?—The Temperature of Baths—Metropolitan or Provincial?—Queen's Nurses in Scotland—Short Items - cxli

**Elementary Anatomy and Surgery for Nurses.** By W. McADAM ECCLES, M.B., B.S., F.R.C.S. V. The Process of Inflammation and the Healing of Wounds - cxliii

**Addenbrooke's Hospital, Cambridge** - cxliii

**Orphans Still Caged** - cxliv

**Notes from Cairo** - cxliv

**Nursing in the West Indies** - cxlv

**Presentations** - cxlv

**Notes and Queries** - cxlv

**The Book World for Women and Nurses** - cxlvi

**Everybody's Opinion** - cxlvi

**Begging Letter Writers—Where to Go** - cxlvi

